# Analysis of knowledge bases and research hotspots of coronavirus from the perspective of mapping knowledge domain

**DOI:** 10.1097/MD.0000000000020378

**Published:** 2020-05-29

**Authors:** Qiulei Jia, Shuqing Shi, Guozhen Yuan, Jingjing Shi, Shuai Shi, Yuanhui Hu

**Affiliations:** a Cardiovascular Department, Guang’anmen Hospital, China Academy of Chinese Medical Sciences; b Graduate School, Beijing University of Chinese Medicine, Beijing, China.

**Keywords:** bibliometric analysis, CiteSpace, coronavirus, research hotspots

## Abstract

**Background:**

Coronaviruses have drawn attention since the beginning of the 21st century. Over the past 17 years, coronaviruses have triggered several outbreaks of epidemic in people, which brought great threats to global public health security. We analyzed the publications on coronavirus with bibliometrics software and qualitatively and quantitatively evaluated the knowledge base and hot topics of coronavirus research from 2003 to 2020.

**Methods:**

We explored the publications on coronavirus in the Web of Science core collection (WOSCC) from 2003 to 2020. Bibliometric analysis, evaluating knowledge base, and research hotspots were performed based on CiteSpace V (Drexel University, Chaomei Chen).

**Results:**

There were a total of 8433 publications of coronavirus. The research on coronavirus boomed when a novel coronavirus triggered outbreaks in people. The leading country was the United States, and the leading institution was the University of Hong Kong. The most productive researchers were: Yuen KY, Drosten C, Baric RS. The keywords analysis showed that SARS-CoV, infection, acute respiratory syndrome, antibody, receptor, and spike protein were research hotspots. The research categories analysis showed that virology, microbiology, veterinary sciences, infectious diseases, and biochemistry and molecular biology were hot research categories.

**Conclusions:**

Bibliometric analysis of the literature shows the research on coronavirus boomed when a novel coronavirus triggered outbreaks in people. With the end of the epidemic, the research tended to be cooling. Virus identification, pathogenesis, and coronavirus-mediated diseases attracted much attention. We must continue studying the viruses after an outbreak ended.

## 1 Introduction

Coronaviruses are members of the subfamily *Coronavirinae* in the family *Coronaviridae* and the order *Nidovirales.* Coronaviruses, in particular, have become a global health threat following the outbreak of severe acute respiratory syndrome (SARS) in 2002 and 2003 in China.^[1]^ In 2012, another coronavirus, Middle East respiratory syndrome coronavirus (MERS-CoV) emerged in Middle Eastern countries,^[2]^ and the outbreak as well as occurred in South Korea in 2015.^[3]^ Both severe acute respiratory syndrome coronavirus (SARS-CoV) and MERS-CoV are highly pathogenic. The emergence and spread of the new coronavirus, named severe acute respiratory syndrome coronavirus-2, across China put coronavirus in the spotlight again.

Before the epidemic of SARS, only a few coronaviruses were known to be circulating in humans, causing only mild respiratory infection,^[4]^ not the highest scientific imperative. Numerous scholars have started to pay attention to the coronavirus research field, and the number of papers related to coronavirus had a surge because of SARS. We adopted a bibliometrics approach to provide an overview of the publications, which were obtained from the Web of Science (Thomson Reuters Company) database from 2003 to 2020 and analyzed the influential countries, institutions, authors, highlight its intellectual basic, research hotspots, and frontier. We hope our results could be helpful to gain complete and accurate information about coronavirus research domains.

## 2 Methods

### 2.1 Data sources and retrieval strategies

Data for the bibliometric analysis was collected from WOSCC. We carried out literature retrieval using index words “coronavirus” in topic search. The time span for the search was from 2003 to 2020 (the retrieved date on February 11, 2020). Citation index was SCI-expanded. Document type was the only article, and language or category was not restricted. All records (include titles, authors, sources, abstracts) and references in our search results were exported in plain text format. The data is all secondary data and does not contain any personal information, so informed consent was not required.

### 2.2 Analysis methods

CiteSpace V (Version 5.3 R4, Drexel university, Chaomei Chen)^[5]^ were very useful in performing bibliometric analysis. CiteSpace V was used to generate the co-authorship network of countries/institutions/authors, the co-occurrence network of category/keywords, and co-citation map of authors/references.

In the co-authorship network map, the nodes represent elements such as countries, institutions, authors. The node size represents the number of publications published by the countries, institutions or author in the WOSCC. The links between nodes represent cooperative relationships. The color of the lines represents the time of the first cooperation between the 2 countries, institutions, or authors. The thickness of the lines is positively correlated to the collaboration between the countries, institutions, or authors.

In the co-occurrence network map, the nodes represent different categories and keywords. The node size in co-occurring research categories network represents the number of publications in each category, and the node size in co-occurring keywords network represents the frequency of occurrence. The links represent co-occurrence relationships. The color of the lines represents the time of the first co-occurring between the 2 categories or keywords. The thickness of the lines is positively correlated to the number of publications with the same label between the categories, or positively related to the frequency of keywords occurring together.

In the co-citation maps, the nodes represent cited authors or references, and the links represent corresponding authors or references that were cited together.^[6,7]^ The color of the connection line indicates the time of the first co-citation. The node size represents the co-citation frequency. The color of the line indicates the time of the first co-citation. The thickness of the lines is positively correlated to the frequency of co-citation between 2 authors or references.

Nodes with purple circles represent studies with high betweenness centrality and are defined as turning points in a bibliometric study.^[8]^ The burst detection function of CiteSpace software was used to measure the sharp increasing of citations of keywords within a short time, helping understand the research frontiers. A time span in a 1-year slice was indicated with a different color in the network links and nodes.

The parameters of CiteSpace were as follows: time slice (2003–2020), year of each slice, term source (all selected), node type (1 at a time), selection criteria (Top50), pruning (Pathfinder), and visualization (cluster view static, display merged network). Selection criteria changed to Top100 when co-cited references were analyzed.

## 3 Results

### 3.1 Publication outputs

**Figure 1:**
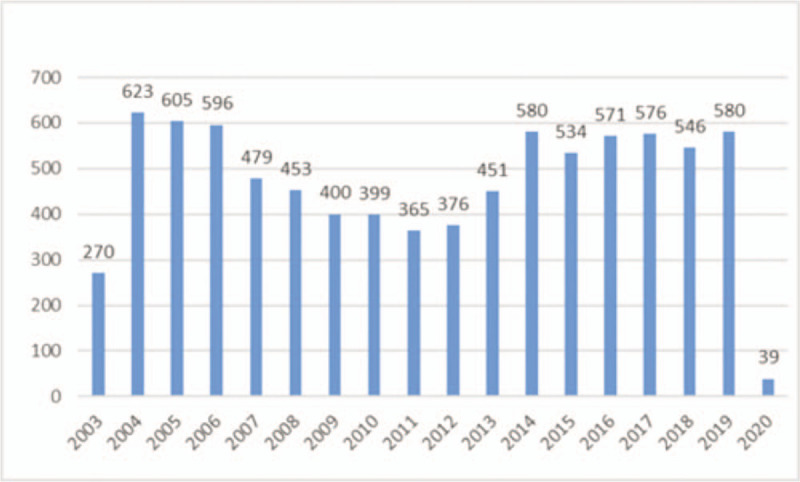
The annual number of publications on coronavirus indexed in WOSCC from 2003 to 2020. The abscissa in the figure represents the year, and the ordinate represents the total number of publications.

Based on the selection criteria, 8433 publications of coronavirus in WOSCC from 2003 to 2020 were indexed. In 2003, 270 articles were published. From 2004 to 2006, the number of publications per year increased to nearly 600. Then, there was a decline in the number of publications per year between 2007 and 2011. After that, the number increased again and gradually rose to the level of 530 to 580 papers per year (Fig. 1).

### 3.2 Distribution of countries and institutions

**Figure 2:**
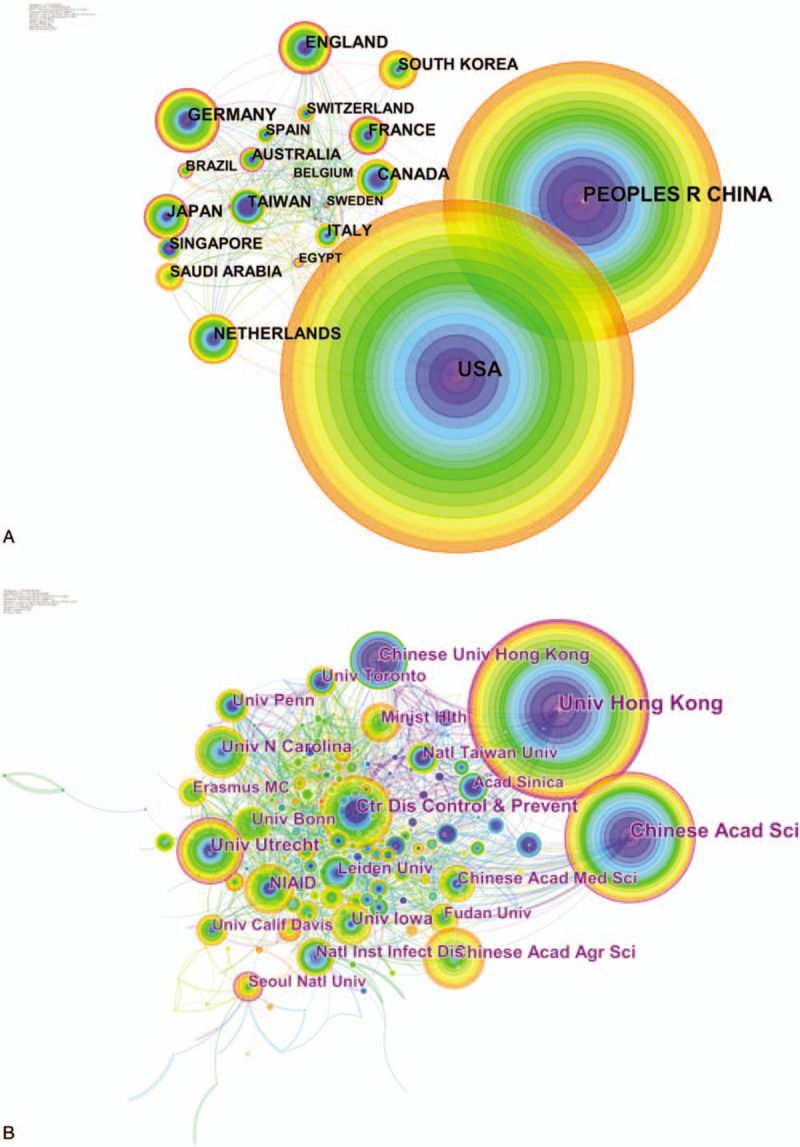
A. Network of countries on coronavirus. The size of nodes represents the number of publications published by the countries/regions. The nodes with purple outer rings are countries with high centrality. The links between nodes represent cooperative relationships between countries. B. Network of co-authors’ institutions on coronavirus. The size of nodes represents the number of publications published by the institutions. The nodes with purple outer rings are institutions with high centrality. The links between nodes represent cooperative relationships between institutions.

A total of 84 countries and regions made contributions to publications on coronavirus research (Table 1, Table 2, Fig. 2A). The USA had the largest number of publications (2791 papers), followed by the People's Republic of China (2231 papers), Germany (564 papers), Netherlands (466 papers) and England (447 papers) were the top 5 prolific countries. Moreover, the centrality which ranked the first was Germany and France (0.16), followed by England (0.15), Brazil (0.15), Sweden (0.13).

**Table 1:**
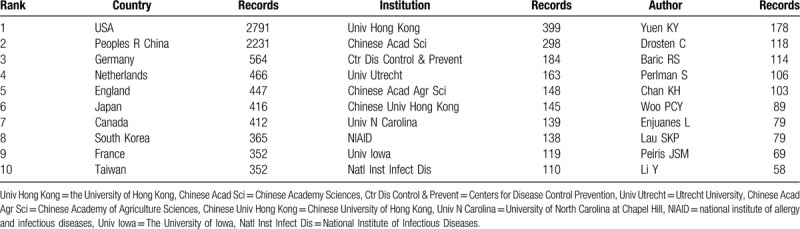
The top 10 countries, institutions, and authors contributed to publications on the coronavirus from 2003 to 2020.

**Table 2:**
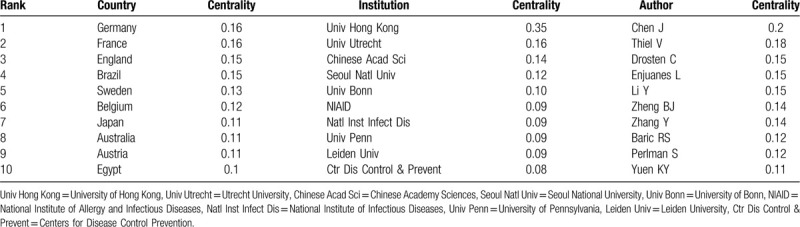
The top 10 countries, institutions, and authors with high centrality value of the coronavirus publications from 2003.

The publications on coronavirus were contributed by 333 institutions (Fig. 2B). As shown in Table 1, the University of Hong Kong (399 papers) had the most productive publications, followed by Chinese Academy Sciences (298 papers), Centers for Disease Control Prevention (184 papers), Utrecht University (163 papers), and Chinese Academy of Agriculture Sciences (148 papers). The centrality which arranged the first was the University of Hong Kong (0.35), followed by Utrecht University (0.16), Chinese Academy Sciences (0.14), Seoul National University (0.12), University of Bonn (0.10) (Table 2). We can evaluate the academic contributions and social activities in coronavirus research by institutions cooperation network. Figure 2B indicates that institutions cooperate with others to some extent.

### 3.3 Distribution of authors and cited authors

**Figure 3:**
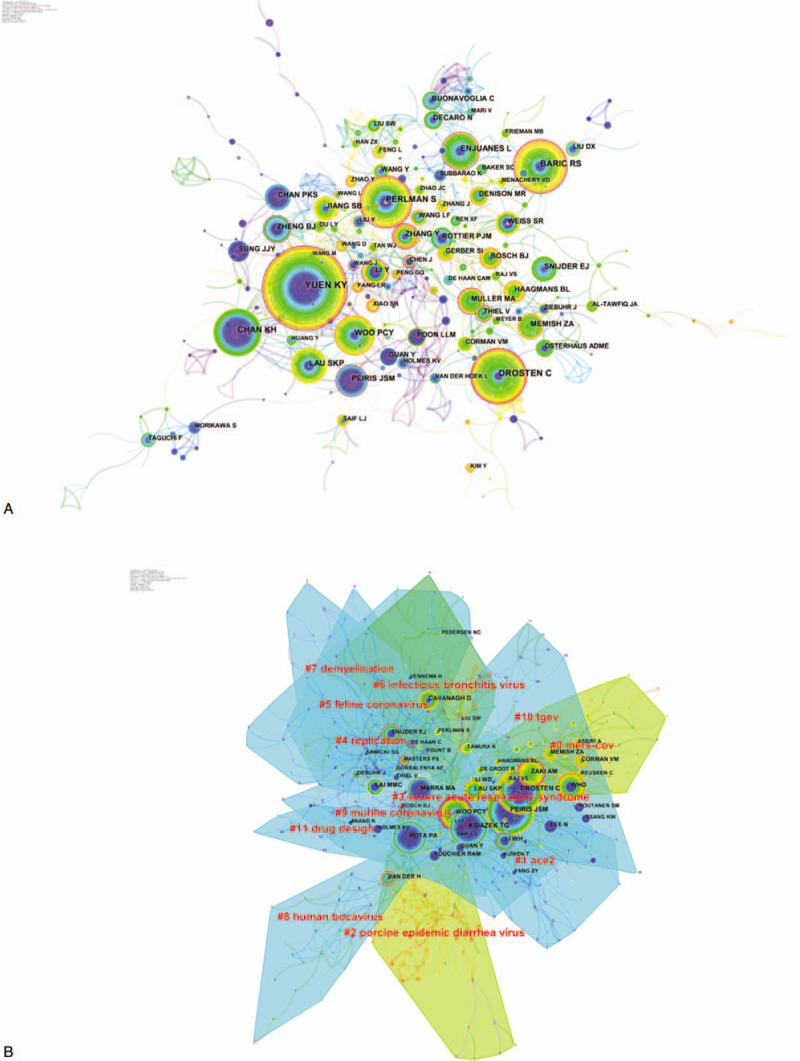
A. Co-authorship network on coronavirus. The size of the nodes represents the number of publications published by the authors. The nodes with purple outer rings are authors with high centrality. The links between nodes represent cooperative relationships between authors. B. Co-citation map of authors on clustered network of coronavirus. A cluster represents the cited authors’ study of similar categories. Clusters are labeled in red text. The size of the nodes represents the frequency of co-citation authors. The nodes with purple outer rings are co-citation authors with high centrality. The links represent the corresponding authors cited together.

Five hundred ninety-one authors contributed to the publications on coronavirus research. The authors who had the most productive (Table 1) were Yuen KY (178 papers), Drosten C (118 papers), Baric RS (114 papers), Perlman S (106 papers), Chan KH (106 papers). What's more, the top-ranked item by centrality (Table 2) was Chen J (0.20), followed by Thiel V (0.18), Drosten C (0.15), Enjuanes L (0.15), Li Y (0.15). The co-authorship map shows that quite a few connection lines are observed between the nodes, indicating that there was a close relationship between the scholars in this field (Fig. 3A).

As can be seen from the co-authorship map, the scholars could be roughly divided into 5 major academic groups:

(1) the critical members of the academic group 1 were Li Y, Zhang Y, and Liu Y, who was mainly engaged in MERS-CoV and its receptor-binding domain ^[9,10]^;
(2) the key members of the academic group 2 were Enjuanes L, Perlman S, and Baric RS, who mainly engaged in the envelope (E) protein in SARS-CoV ^[11,12]^;
(3) the leading members of the academic group 3 were Yuen KY, Peiris JSM, Chan KH, and Lau SKP, who mainly engaged in isolation and identification of a novel coronavirus ^[13–15]^;
(4) the leading members of academic group 4 included Thiel V, Ziebuhr J, and Snijder EJ, who mainly engaged in research on coronavirus replication ^[16,17]^;
(5) the leading members of academic group 5 included Bosch BJ, Muller MA, Meyer B, and Corman VM, who mainly engaged in research on the antibody of coronaviruses.^[18,19]^

Co-cited authors mean that the authors are cited together by the same paper. Table 3 presents the top 10 cited authors. Among them, Peiris JSM (1519 citations) ranked first, followed by Drosten C (1516 citations), Ksiazek TG (1243 citations), Rota PA (1089 citations), and Woo PCY (1038 citations). The top 5 co-cited authors in terms of centrality were Woo PCY (0.23), De Haan C (0.21), Bosch BJ (0.19), Li WH (0.18), Van Der H (0.17) (Table 4). In the author co-citation cluster map (Fig. 3B), there were 535 nodes, 2537 links, and 12 co-cited author clusters. The silhouette value of clusters #0 to 11 was from 0.756 to 0.973, showing good homogeneity.^[6]^ All clusters were labeled by index terms extracted from the keywords. Nodes of different colors indicate different clusters, and the cluster labels represent the cited authors’ study of similar categories. Authors’ research category in Cluster #0 is MERS. Besides, WHO and Zaki AM were the most significant influential authors in this category. The authors’ research category in Cluster #1 is angiotensin-converting enzyme 2 (ACE2), 1 of the receptors of coronavirus, and Li WH is the most critical scholar in this field. Authors’ research category in Cluster #2 is porcine epidemic diarrhea virus, the articles written by Song D were cited the most times. The authors’ research category in Cluster #3 is SARS, Peiris JSM, Drosten C, Ksiazek TG, and Woo PCY are the important authors in the field of SARS research. The authors’ research category in Cluster# 4 is virus replication, Lai MMC, Snijder EJ, and Ziebuhr J are the leading researchers. A summarization of the author co-citation cluster is provided in Table 5.

**Table 3:**
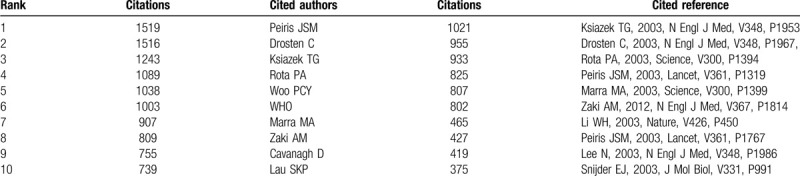
The top 10 cited author and cited reference contributed to publications on the coronavirus from 2003 to 2020.

**Table 4:**
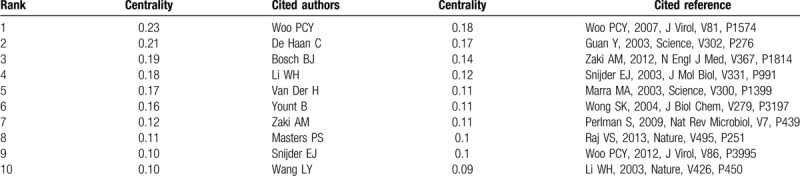
The top 10 cited authors and cited references with high centrality value of the coronavirus publications from 2003 to 2020.

**Table 5:**
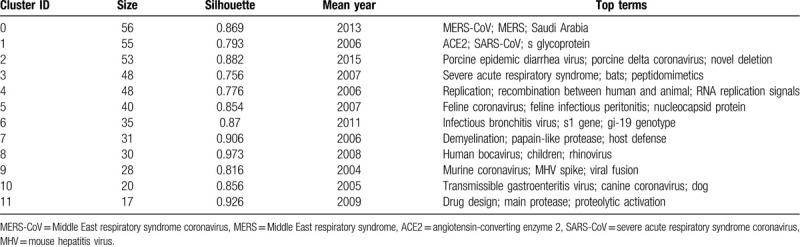
The summary co-cited author clusters on coronavirus.

### 3.4 Distribution of cited references

Two references that were cited at least twice by the same paper were considered to be co-cited. Review references can trace the intellectual roots of researches. Reference co-citation analysis reflects the intellectual structure of coronavirus. A total of 121,296 references were cited in 8443 articles. The citation counts and centrality values of references were shown in Table 3 and Table 4. The top-ranked item by citation counts was Ksiazek TG (1021 citations), who published his output in N Engl J Med in 2003. The reference with the largest centrality was Woo PCY (0.18) published in J Virol in 2007.

**Figure 4:**
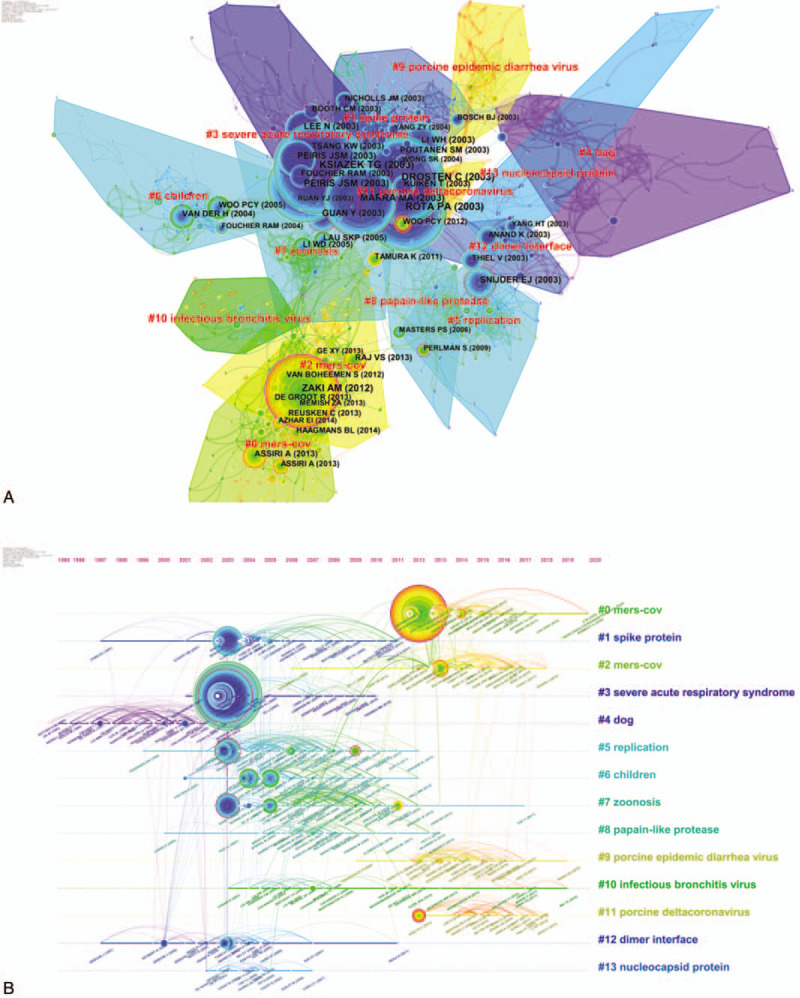
A. Co-citation map of references on cluster network of coronavirus. A cluster represents the cited references in similar categories. Clusters are labeled in red text. The size of the nodes represents the frequency of co-citation references. The nodes with purple outer rings are co-citation references with high centrality. The links represent references cited together. B. Timeline view of co-citation reference clusters. Major clusters are labeled on the right. The clusters are vertically arranged in descending order based on their sizes. The full lines with different colors show the beginning and ending time of clusters. The nodes on the full lines represent cited references, and the links represent references that were cited together. The density of nodes at different time points can reflect the dynamics of the corresponding cluster in the timeline.

In the co-citation network, articles in similarities are commonly cited together and tended to concentrate on 1. The co-citation network was composed of 843 nodes, 3111 links, and 14 co-cited reference clusters (Fig. 4A). The silhouette value of Clusters #0 to 13 was from 0.791 to 0.966, showing good homogeneity. All clusters were labelled by index terms extracted from the keywords. Figure 4B is the timeline view for reference co-citation clusters, which is used to detect and identify changes in development trends in different periods. The full lines with different colors show the beginning and ending time of clusters. The density of nodes at different time points can reflect the dynamics of the corresponding cluster in the timeline. Because only considering the top keyword for each cluster may be insufficient, we have provided more keywords in Table 6.

**Table 6:**
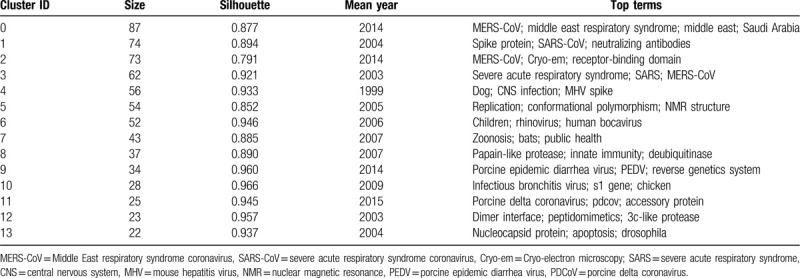
The summary co-cited reference clusters on coronavirus.

According to Table 6 and relevant documents, Cluster #0 consists of 87 co-cited articles relating to MERS-CoV, which had a sustained period since 2012, and a larger number of publications were cited from 2012 to 2017. While Cluster #2 consisting of 73 co-cited articles is named the same the label “MERS-CoV”, the studies were more active from 2012 to 2018. According to the references, Cluster #0 MERS-CoV is more relevant to the clinical features and epidemiology research. In the study of Zaki AM et al,^[2]^ an unknown coronavirus was isolated from the sputum of a 60-year-old man who presented with acute pneumonia in Saudi Arabia, and the novel virus named MERS-CoV. By contrast, Cluster #2 MERS-CoV is more relevant to the research on the structure of MERS-CoV, receptor-binding domain.^[20]^ Cluster #1 is about spike (S) proteins of coronaviruses, associated with cellular receptors to mediate infection of their target cells. ACE2 is a functional receptor for SARS-CoV, which can efficiently bind the S1 domain of the SARS-CoV S protein.^[21]^ The studies in cluster #1 were most active in 2003 to 2010. Cluster #3 is related to the epidemic, clinical features, diagnosis, and prevention about SARS, which sustained for 8 years from 2002 to 2010. Cluster #4 is the earliest cluster, which sustained for 8 years from 1995 to 2003, relating to genotype, the pathogenesis of the virus, which is the basis for the following research on the virus. Cluster #5 is mainly about the replication of SARS-CoV, of which clustering time was from 1999 to 2012, and the studies were most active in 2003 to 2009. Cluster #6, with a larger number of publications from 2004 to 2010, contains the documents reported the other respiratory coronavirus, such as HCoV-NL63^[22]^ and Human bocavirus,^[23]^ infecting children easily. Cluster #7 is mainly about animal coronaviruses, of which clustering time was from 2003 to 2017, and the studies were most active in 2003 to 2013. Cluster #8 is mainly about coronavirus protein structure, having a period of 13 years from 2000 to 2013. Cluster #9 is mainly about the porcine epidemic diarrhea virus (PEDV), which causes acute diarrhea and dehydration in pigs, and the studies were most active in 2012 to 2018. Cluster #10 is related to infectious bronchitis viruses (IBV), isolated from outbreaks in chickens in China. The studies in cluster #10 have been active since 2003, and more active in 2003 to 2008. Cluster #11 is about porcine delta coronavirus (PDCOV), a delta coronavirus detected in feces and intestine samples from pigs with diarrheal disease in 2014, of which clustering time was from 2012 to 2018. Cluster #12 is about the structure of the main coronavirus proteinase,^[24,25]^ with a larger number of publications around 2003. Cluster #13 is about the gene of coronaviruses protein,^[26,27]^ which is short-lived with an associated period of 4 years from 2003 to 2007.

### 3.5 Distribution of research categories

**Figure 5:**
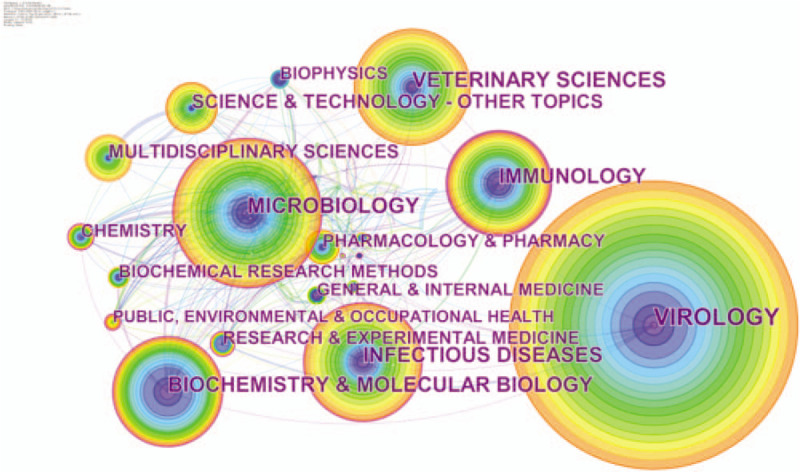
Co-occurring research categories network on coronavirus. The node size represents the number of publications in each category. The nodes with purple outer rings are research categories with high centrality. The links represent co-occurrence relationships.

The publications on coronavirus research in the WOSCC can be attributed to 1 or more categories. Researchers can quickly locate the studies of concern through the distribution of categories analysis. A total of 106 research categories were identified. We analyzed these categories to reveal the high-impact categories in the field of coronavirus. Figure 5 shows that interdisciplinary research is common. Table 7 shows the categories of publications on coronavirus research. The category with the most articles was virology (2636 papers), followed by microbiology (1402 papers), veterinary sciences (1126 papers), infectious diseases (1094 papers), and biochemistry and molecular biology (1058 papers). Besides, the top 5 categories with high centrality were immunology (0.27), biochemistry and molecular biology (0.17), research and experimental medicine (0.17), public, environmental and occupational health (0.16) and microbiology (0.13) (Table 8).

**Table 7:**
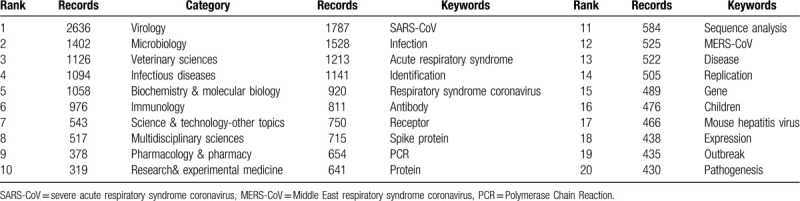
The top 10 research categories and top 20 keywords on coronavirus.

**Table 8:**
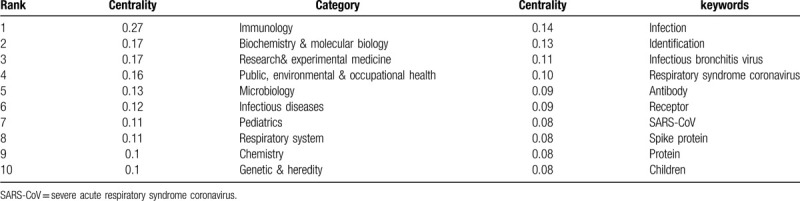
The top 10 research categories and keywords with high centrality value on coronavirus.

### 3.6 Distribution of keywords

The keywords can be considered one of the most important points of a publication and co-keywords analysis can reflect research hotspots, and monitor research frontier transitions.^[28]^ We excluded keywords without meaningful information such as coronavirus and virus, then 132 keywords were obtained.

As is presented in Table 7, the top 5 keywords in terms of citation counts for coronavirus research were listed in order as follows: “SARS-CoV (1781 records)”, “infection (1528 records)”, “acute respiratory syndrome (1213 records)”, “identification (1141 records)” and “respiratory syndrome coronavirus (920 records)”. The top 5 keywords with high centrality were “infection (0.14)”, “identification (0.13)”, “infectious bronchitis virus (0.11)”, “respiratory syndrome coronavirus (0.10)”, “antibody (0.09)”.

**Figure 6:**
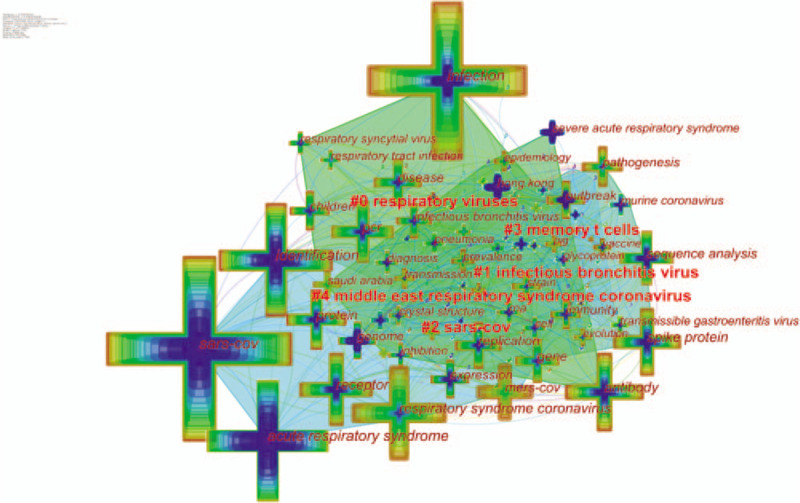
Co-occurring map of keywords on clustered network of coronavirus. A cluster represents the keywords in similar categories. Clusters are labeled in red text. The node size represents the frequency of occurrence. The nodes with purple outer rings are keywords with high centrality. The links represent co-occurrence relationships.

The keyword-term cluster view (Fig. 6) consists of 133 nodes and 529 links, generating 5 keyword clusters. The silhouette value of clusters #0 to 4 was from 0.669 to 0.76, showing good homogeneity. All clusters were labelled by index terms extracted from the keywords. The specific information for the 5 clusters is shown in Table 9. Cluster #0 was mainly about respiratory viruses, which illustrated viral respiratory infections from the angle of the clinic. Cluster #1 was mostly about the genetic aspects of various coronaviruses. Cluster #2 was mainly about SARS-CoV. Cluster #3 was mainly about immunity. Cluster #4 was mostly about MERS-CoV.

**Table 9:**
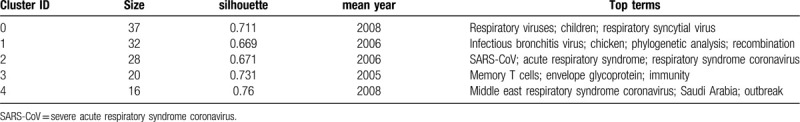
The summary co-occurring keyword clusters on coronavirus.

**Figure 7:**
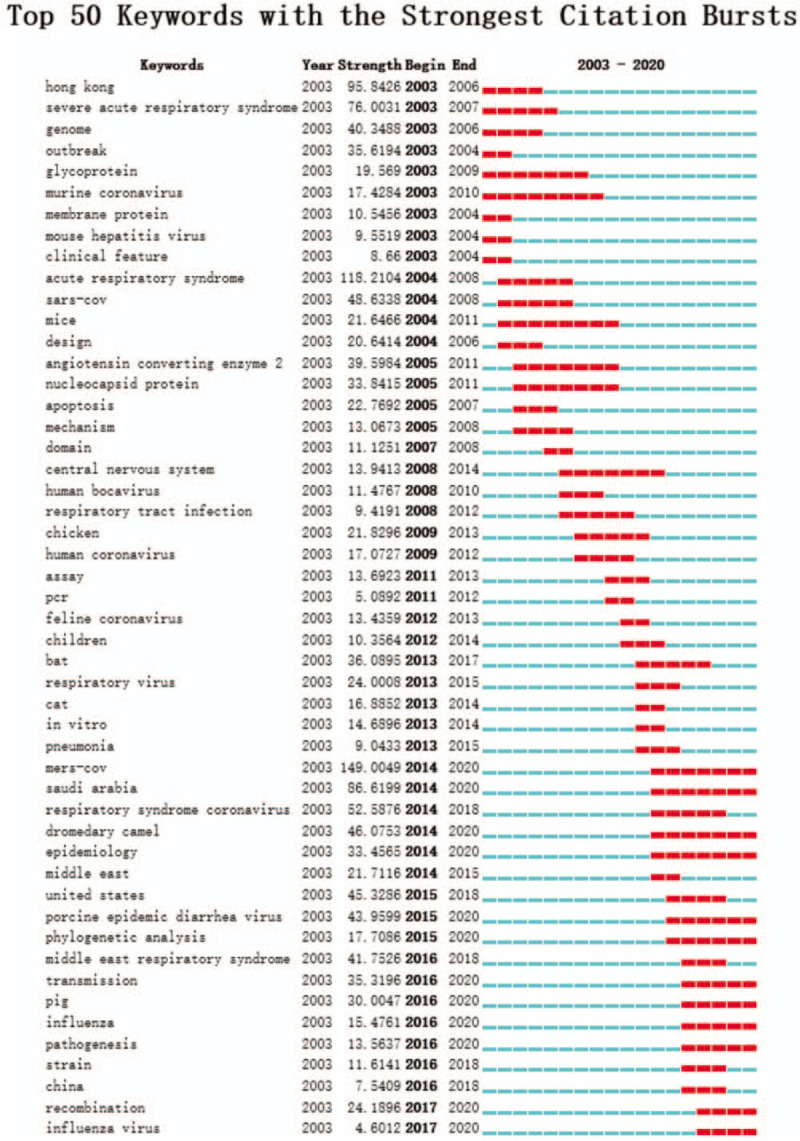
Top 50 Keywords with the strongest citation bursts. The red bars represent some references cited frequently; the green bars represent references cited infrequently.

Keyword-term burst detection is considered as an indicator of research frontiers or emerging trends over time. Figure 7 shows the top 50 keyword terms with the strongest frequency bursts. The keywords citation bursts after 2016 were listed as follows: transmission (35.3196), pig (30.0047), influenza (15.4761), influenza virus (4.6012), recombination (24.1896), and pathogenesis (13.5637), which are the frontiers of research in the coronavirus. Researchers may consider these directions for future studies.

## 4 Discussion

### 4.1 General data

The number of articles that included the word “coronavirus” in term jumped dramatically after the SARS outbreak in 2003. Nevertheless, when concerns faded as the outbreak was contained, the number of publication outputs per year decreased. There were just 365 papers published on coronaviruses in 2011. With the MERS outbreak, the quality of publications on coronavirus gradually increased again from 2012. The number of publications output per year has been stable since the emergence of MERS, but it never reached the post-SARS highest level. Research on coronavirus has a close relationship with infectious diseases outbreaks coronavirus triggered in people.

### 4.2 Co-authorship: countries, institutions, and authors

The analyses of distributions in countries and institutions could help in promoting teamwork and global co-operation in this field. The top 10 countries/regions were located in North America (the USA and Canada), Asia (China, Japan, South Korea, and Taiwan), and Europe (Germany, Netherlands, England, and France). The United States had the greatest number of published articles, reflecting its dominant position in coronavirus research. China ranked second, and Taiwan ranked 10th, which was associated with the nationwide epidemics of SARS. Perhaps, the prevention, control, and treatment of the coronavirus disease 2019 (COVID-19) outbreak will also depend on research and cooperation between China and the United States to achieve breakthroughs. While South Korea ranked 8th, and most articles published in 2016 to 2019 after the MERS outbreak. The cooperation between European countries was closed. Among the 4 European countries, Germany, England, and France had a higher value of centrality, indicating they played a ligament in the cooperative network between countries.

The top 10 institutions were located in China (Univ Hong Kong, Chinese Acad Sci, Chinese Acad Agr Sci, Chinese Univ Hong Kong), USA (Ctr Dis Control & Prevent, Univ N Carolina, NIAID, Univ Iowa), Netherlands (Univ Utrecht), and Japan (Natl Inst Infect Dis). The global distribution of institutions was consistent with the distribution of countries. Lots of links between institutions in the network reflect the active cooperation of these institutions. The most prolific institutions were the University of Hong Kong and Chinese Academy Sciences, in terms of centrality, the University of Hong Kong was the critical institutions of studying coronavirus. We speculated that the possibility of this phenomenon was: the University of Hong Kong has strong scientific research ability; SARS emerged in Guangdong China first, as well as Hong Kong was hard hit with high morbidity and mortality.

Among the top 10 productive authors, 5 were from the University of Hong Kong (Yuen KY, Chan KH, Woo PCY, Lau SKP, Peiris JSM), Drosten C was from University of Bonn in Germany, Baric RS was from University of North Carolina in the USA, and Perlman S was from University of Iowa in the USA. Enjuanes L was from Consejo Superior de Investigaciones Científicas (CSIC) in Spain. Li Y was from the Chinese Academy of Sciences in China. They and their institutions were the main research forces in this area. The collaboration analysis indicated broad cooperation among researchers all over the world. From the view of centrality, Chen J was the author with the largest centrality, what's more, Drosten C, Enjuanes L, Li Y, Baric RS, Perlman S, Yuen KY were included in the list of the top 10 productive authors. The centrality of them was all higher than 0.1, indicating they were the most vigorous and vital scholar in the field. It can be seen that Hong Kong owns a significant contribution and a considerable influence on coronavirus research, which is likely to produce more important academic achievements in this field.

### 4.3 Co-citation: references and authors

Levels of authors’ influence are reflected by co-citation analysis. Peiris JSM, Drosten C, Woo PCY, and Lau SKP were both highly cited authors and productivity authors, moreover, Woo PCY owned the highest centrality. So the papers written by Woo PCY meant high quality and hugely influential. Woo PCY mainly engaged in research on the nucleocapsid protein and spike polypeptide of SARS-CoV,^[29–31]^ also discovered and isolated a novel coronavirus, coronavirus HKU1, from patients with pneumonia.^[32]^ Cluster #0 MERS-CoV contained the largest author group, who illustrated the transmission and epidemic of MERS-CoV in the middle east,^[33]^ and clinical observation of MERS.^[34]^

Reference co-citation analysis can be used to identify the knowledge base. The knowledge base of coronavirus is divided into 2 types: early founding documents of coronavirus; highly cited and high centrality references. From the references-cited timeline map, we can obtain the evolution of the knowledge base.

The mean year of cluster#4 is 1999, the studies in cluster#4 relating to genotype, structure protein, and pathogenesis of virus, which is the basis for the following research on the virus. The starting and ending time of cluster#0, #2, #3, #6, #9, #10, #11 is in accord with the epidemic of coronaviruses related to infectious diseases. With the plague over, the researches were no longer boom.

In studies of Peiris JSM et al,^[35]^ researchers first supported the clinical presentation and risk factors associated with severe disease and isolated viruses from 2 patients. By the use of serological and reverse transcription-polymerase chain reaction, the virus was found belonging to the family *Coronaviridae*. The top 2 cited references were published in 2003 by Ksiazek TG^[36]^ and Drosten C^[37]^ respectively. They isolated a novel coronavirus from SARS patients and verified that the virus had an etiologic role in SARS. Another 2 highly cited references published in 2003 by Rota PA^[38]^ and Marra MA^[39]^ reported the genome sequence of the SARS-CoV respectively. The paper published in 2003 by Li WH et al^[21]^ reported that ACE2 is a functional receptor for SARS-CoV. Wong SK et al^[40]^ demonstrated that a 193-amino acid fragment of the S protein (residues 318–510) bound ACE2 more efficiently than did the full S1 domain (residues 12–672). These research findings played significant roles in blocking viral replication and facilitated the development of intervention strategies. Another highly cited reference published in 2012 by Zaki AM et al^[2]^ reported the isolation of MERS-CoV from a man with pneumonia in Saudi Arabia. Raj VS et al^[20]^ identified dipeptidyl peptidase 4 (DPP4) as a functional receptor for HCoV-EMC.

The paper with the high centrality value published in 2003 by Guan Y et al^[41]^ reported that a closely related SARS-like virus was isolated from wild animals found in a live-animal market in Guangdong, China, suggesting that the markets provide a venue for the animal SARS-like virus to amplify and to be transmitted to new hosts, including humans, and this is critically important for public health. The paper with the highest centrality value was published in 2007 by Woo PCY et al^[42]^ reported that 3 novel coronaviruses bat-CoV HKU4, bat-CoV HKU5, and bat-CoV HKU9 belong to the different subgroup of group 2 coronavirus by complete genome sequencing. And the 4 subgroups of group 2 coronaviruses probably originated from a common ancestor. Snijder EJ et al^[43]^ concluded that SARS-CoV is distantly related to group 2 coronaviruses. The SARS-CoV genome lacks the genes that are common in group 2 viruses, like papain-like proteinase 1 and cyclic phosphodiesterase like and HE genes. However, it encodes some unique protein sequences, underlining the ability of coronaviruses to the gross evolution, helping people have a new understanding of the mechanism of coronavirus RNA synthesis. Another high centrality value paper published in 2012 by Woo PCY et al^[44]^ reported 7 novel deltacoronaviruses isolated from mammalian and avian. Furthermore, they proposed that bats and birds are ideal hosts for the coronavirus gene source, bats for Alphacoronavirus and Betacoronavirus and birds for Gammacoronavirus and Deltacoronavirus, to fuel coronavirus evolution and dissemination.

These critical documents provide theoretical support for the research on coronavirus. We can grasp the subject framework and the knowledge base containing the transmission, clinical presentation, isolation SARS-CoV and MERS-CoV, complete sequencing of the virus genome, the functional receptor for the SARS-CoV and MERS-CoV, SARS-CoV RNA synthesis and virus replication, group and subgroup features and the gene source of novel coronaviruses.

### 4.4 Hotspot analysis

#### 4.4.1 Research categories analysis

Research categories analysis revealed the hot categories and the confluence of different disciplines. The categories listed in Tables 7 and 8 mainly involve basic medical sciences (virology, microbiology, biochemistry & molecular biology, immunology, pharmacology, & pharmacy), clinical medicine (infectious diseases, pediatrics, respiratory system), veterinary sciences, and public health (public, environmental, and occupational health). Both humans and animals are vulnerable to coronaviruses, which usually bring about respiratory system infection.^[45]^ Children are especially vulnerable to be infected. Molecular biology techniques such as PCR and genome sequencing apply to coronavirus research. The research and development of drugs against coronaviruses have made advances. The category with the highest centrality value was immunology, which plays an essential role in the development of coronavirus research. Undoubtedly, virus pathogenesis, antiviral immunity, and vaccine development are closely related to immunology. Basic medical sciences provide a support function for research on coronavirus. Coronaviruses can spread across species. Moreover, SARS and COVID-19 are highly contagious diseases, so coronavirus is of considerable significance on public health.

#### 4.4.2 Keywords analysis

Keywords analysis illustrates the development of a research topic more comprehensively.^[5]^ The high frequency and high centrality keywords obtained before are categorized as 2 research hotspots and list accordingly.

### 4.5 Coronavirus-mediated diseases

SARS-CoV caused global outbreaks of a severe acute respiratory disease in 2002 to 2003, and the overall fatality rate was nearly 15%.^[1]^ Besides fever and respiratory symptoms, SARS-CoV also caused systemic disease, with evidence of infection of the gastrointestinal tract, liver, kidney, and brain, among other tissues.^[46]^ The epidemic was controlled and ended within 8 months. Ten years later, since the emergence of MERS-CoV in Saudi Arabia in 2012, this virus caused 2 severe respiratory syndrome outbreaks. One short-lived outbreak occurred in South Korea in 2015, and the other epidemic that has persisted in Saudi Arabia since 2013.^[3,47]^ According to the WHO, the fatality rate of MERS was 34.4% by November 2019.^[48]^ Therefore the researches on MERS-CoV are continuing now, while very few studies have focused on SARS.

Acute respiratory infections are a major cause of morbidity in children both in developed and developing countries. HCoV-OC43 discovered before the SARS was recognized as an important cause of upper respiratory tract infections.^[45]^ Following the discovery of SARS-CoV, coronaviruses NL63 (HCoV-NL63) was identified in 2004.^[49]^ It is reported that OC43 and NL63 are detected at a younger age and more frequently in the pediatric population.^[50]^ A study reported that HCoV-NL63 was undergoing a continuous mutation and had the potential to cause severe lower respiratory disease in children.^[51]^

Coronaviruses also could cause life-threatening diseases in the animal. IBV, a cause of severe upper respiratory tract and kidney disease in chickens, which poses a significant economic threat to the poultry industry.

### 4.6 The pathogenesis of coronaviruses

The pathogenesis of SARS is complex. In this field, virus receptors and entry into host cells are the hotspots. Spike protein as one of the most critical surface glycoproteins of SARS and MERS-CoV is necessary for virus attachment on susceptible cells. It represents a key target for developing therapeutics to block viral entry and inhibit membrane fusion.^[52,53]^ SARS-CoV spike protein uses a mechanism similar to that of class 1 fusion proteins in mediating membrane fusion.^[54]^ The host functional receptor for SARS-CoV was identified as ACE2, a metallopeptidase that is expressed on many human organ tissues.^[21]^ The entry of MERS-CoV is mediated by DPP4,^[55]^ which is plentiful in the respiratory tract of humans including the bronchial mucosa but is also expressed in a variety of tissues such as bronchial epithelial cells and kidney cells.^[56]^ MERS-CoV was the only coronavirus shown to use DPP4 to gain entry to host cells.^[57]^ Once inside the cell, transcription in the cytoplasm and RNA replication in the cytoplasm proceed.

## 5 Conclusions

In this study, we investigated the knowledge progress of the coronavirus domain by literature mining strategies, which can help us understand the evolution and trends in the coronavirus field visually. The result graphics show that the research on coronavirus was boom when a novel coronavirus triggered outbreaks in people. With the end of the epidemic, the research tended a cold. Virus identification, pathogenesis and coronavirus-mediated diseases attracted much attention. We must continue studying the viruses after an outbreak ended.

## 6 Limitations

However, this study also has some limitations. First, the data were retrieved from WOSCC and only contained research papers (as opposed to reviews, meeting abstract, etc). Second, we only read English language papers because of the language barrier, which could have neglected some key researches published on coronavirus.

## Author contributions

**Conceptualization:** Qiulei Jia, Shuqing Shi

**Data curation:** Qiulei Jia, Shuai Shi

**Formal analysis:** Qiulei Jia, Shuqing Shi

**Methodology:** Qiulei Jia, Guozhen Yuan, Jingjing Shi.

**Project administration:** Yuanhui Hu.

**Writing – original draft:** Qiulei Jia.

**Writing – review & editing:** Qiulei Jia, Shuqing Shi.
